# The Relationship between Ozone and Human Blood in the Course of a Well-Controlled, Mild, and Transitory Oxidative Eustress

**DOI:** 10.3390/antiox10121946

**Published:** 2021-12-04

**Authors:** Gerardo Tricarico, Valter Travagli

**Affiliations:** 1 Presidio Ospedaliero Sant’Andrea, 13100 Vercelli, Italy; gerardo.tricarico@aslvc.piemonte.it; 2 UCM United Campus of Malta, Higher Education Institution Foundation, MSD 2080 Msida, Malta; 3 Dipartimento di Biotecnologie, Chimica e Farmacia—Dipartimento di Eccellenza Nazionale 2018–2022, Università degli Studi di Siena, 53100 Siena, Italy

**Keywords:** oxidative therapies, oxidative stress, ozone therapy, blood, reactive oxidative species, lipid ozonation products, immunomodulation

## Abstract

In the last twenty years there has been a proliferation of articles on the therapeutic use of ozone. As it is well-known, the term ozone therapy is very broad. It ranges from either systemic or loco-regional administration of unstable gaseous oxygen/ozone mixtures to the topical application of stable ozonated derivatives. Anyway, in relation to the absence of specific receptors and the extreme reactivity with the biological liquids with which it comes into contact, gaseous ozone cannot be classified as either a drug or a pro-drug. When the gaseous ozone impacts a biological matrix, both reactive oxygen species (ROS) and lipid oxidation products (LOPs) are formed. They represent the effector molecules responsible for modulating the therapeutic activity in the body. Apart from the merits of the action mechanisms resulting from the use of ozone, this article seeks to validate the practice of ozone therapy as an adjuvant treatment in full compliance with the physiology of the whole organism.

## 1. Introduction

The organism of higher animals relates to the stability of the internal cell milieu with many daily rhythms of the body, such as temperature, secretion of hormones and numerous others [1].

Over time, the importance of the organism adapting to various stimuli has become increasingly evident together with the importance of the response of each organism in relation to its own specificity in terms of the search for or maintenance of balance and functional integrity [2].

In such a context, allostasis represents a more accurate concept of homeostasis (remaining stable by staying the same). Based on the multi-point property of allostasis, we could imagine the allostatic system as a special “buffering system” [3].

The term “stress” refers to an event or series of events that evoke a set of physiological responses. Targeted oxidative stress is well described as “oxidative eustress” and it elicits adaptive responses in cells. On the other hand, “oxidative distress” is the result of chronic, uncontrolled overproduction of noxious metabolites that can result in tissue damage [4].

Such opposing effects are expected due to the differences in temporal (acute vs. chronic) and spatial (subcellular setting vs. bloodstream) formation of reactive species. Both the correct use and dosage of chemical agents with pro-oxidant action are the basis of therapeutic approaches characterized by a paradoxical effect on the organism. A well-controlled, mild, and transitory eustress could provoke a healing reaction in a diseased organism.

**Figure 1:**
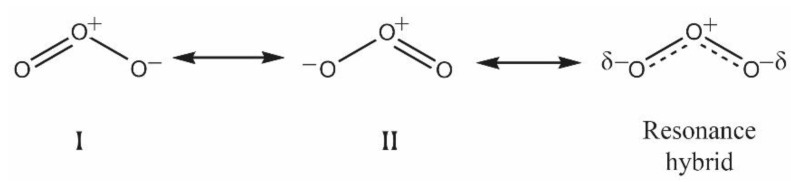
Resonance structures of the ozone molecule.

Ozone under normal conditions is a very unstable molecule and can be represented by three interchangeable formulae (Figure 1). Ozone is a polar molecule with a dipole moment of 0.5337 D. As for symmetry, it shows a bent structure similar to the water molecule, with the O—O—O bond angle at 116.78° [5].

Its maximum electrochemical potential in the gas phase is E° = +2.07 V [6]. The consequences of this intense electric charge mean that ozone takes part in inorganic reactions as an exceptionally powerful oxidizing agent. On the other hand, the simple contact of a gaseous mixture of oxygen/ozone with a biological liquid (e.g., blood, saliva, exudate, transudate) causes a reaction with organic compounds and the formation of peroxide compounds and/or addition to double bonds with the formation of ozonides, in many cases only as intermediates. These data, which are reasonable from a theoretical point of view, remain difficult to analyze and measure due to the complex nature of the reaction types. The ozone derivatives that are formed by a chemical reaction at the level of water-soluble compounds and lipid unsaturation are in turn equipped with an electric charge with an amplitude strong enough to generate a persistent perturbation of the electromagnetic field, with consequent rapid interaction with the cell’s own electric field, its cytoplasmic components, and the tissues of which they are components [7,8]. Irrespective of these facts, gaseous ozone is actually a very toxic substance for all living creatures, but as with all the other substances used in pharmacology, this toxicity is also the principle on which its therapeutic effects are based. The ozone toxicity for the respiratory system remains true even when small amounts of ozone (sub-ppm level) are inspired for days or months. This is because about 70 m^2^ of the alveolar human surface is unprotected by a minimal layer of lining fluid and therefore lacks sufficient antioxidant protection [9].

Despite these aspects, the term “ozone therapy” includes multiple ways of using ozone or its derivatives for therapeutic purposes [10]. After the invention of the first medical ozone generator by the physicist Joachim Hänsler (1908–1981), the physician Hans Wolff (1927–1980) deserves the credit for having developed the ozonated autohemotherapy by insufflating ex vivo a gas mixture composed of at most 5% ozone and the remaining part O_2_ into blood contained in a sterile ozone-resistant glass bottle [11]. For completeness’ sake, this treatment is inspired by the hematogenous oxidation therapy (HOT) according to Wehrli and the ultraviolet autohemoradiation (UVB) according to Wiesner in the case of extracorporeal blood mixed with oxygen under UV radiation and its reinfusion in the same patient [12]. Until the 80s, ozone therapy was used frequently in Germany in an empirical way without knowing the mechanisms of action of ozone dissolved in human blood. Between 1990 and today many studies of prof. Bocci [13,14,15,16,17,18,19,20,21,22,23,24,25] and other researchers [26,27,28,29,30,31] have elucidated the biochemical, immunological, and molecular mechanisms of action, of non-toxic ozone therapy, non-toxic due to the methods of clinical application [32,33]. In fact, in the case of ozone therapy, its toxicity becomes nil because related to both a calibrated dose and, above all, the interaction with the powerful antioxidant system of the blood in a reproducible and well-controlled protocol. The aim of this paper is to appraise the molecular basis of autohemotherapy, a treatment that involves drawing the patient’s blood and re-administering it back into the patient after the contact with an appropriate dose of a gaseous mixture of oxygen-ozone under clearly defined conditions. For international homogeneity requirements, we will continue to use the term major-autohemotherapy and its acronym M-AHT in the case of intravenous reinfusion of ozone-treated blood, although the definition of systemic intravenous therapy by ex vivo ozonation would be the most accurate and appropriate [34].

## 2. Background

Blood is a liquid tissue composed of about 55% plasma and 45% cells, mostly erythrocytes, able to cooperate in reducing the strong oxidant properties of a minimal ozone dose. The plasma, in comparison to the alveolar lining fluid [9] has a wealth of hydrophilic reductants, such as uric acid (about 400 μM), ascorbic acid (about 50 μM), a small amount of glutathione (about 3.4 µM). It also contains about 570 µM of albumin, which has a protective cysteine in position 34 and eleven nucleophilic groups [35]. Moreover, the presence of transferrin and ceruloplasmin quenches oxidizing reactions by chelating transition metals (Fe^2+^ and Cu^+^), which in the presence of hydrogen peroxide, via the Fenton’s reaction, or in the presence of the anion superoxide, via the Haber–Weiss reaction, will catalyze the formation of the highly reactive hydroxyl radical. There is a great difference when ozone, according to Henry law, equilibrates in absolutely pure water proportionate to its concentration and shows a half-life of 110 h at +5 °C [6], or when ozone, dissolved in the blood or in other fluids immediately reacts with either ions or biomolecules, among which polyunsaturated fatty acids (PUFA), proteins, carbohydrates and possibly RNA and DNA [36]. In such a case, ozone reacts coming back to the form of molecular oxygen leading to the formation of reactive oxygen species (ROS), lipid oxidation products (LOPs) and to a variable percentage of oxidized antioxidants [37].

Hydrogen peroxide as ROS is a natural oxidant and, as for its unionized form, it freely diffuses into all the blood cells, mainly erythrocytes, eliciting a significant number of biochemical reactions [38]. In fact, the formation of a transient and dynamic gradient between the plasma and the cytoplasmic water of blood cells makes this oxidant a very early effector. The absence of toxicity is guaranteed by the ozone dosage and by the blood ozonation conditions adopted. In fact, the internal cell environment contains an abundance of GSH, thioredoxin, catalase and GSH-peroxidase as neutralizing agents. Moreover, ROS are produced only during the ex vivo procedure and they are not more present during the reinfusion of ozonated blood in the donor [39]. On the other hand, the ROS biology in blood after ozonation is a very complex phenomenon and recent acquisition in terms of reactive sulfur species as new reactive oxygen species could be of vital importance to human health issues [40].

**Figure 2:**
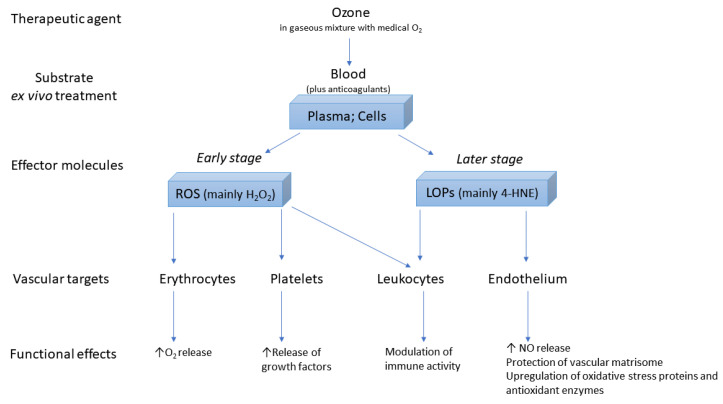
Schematic representation of the key molecular mechanisms of action of the ozone on the blood and intravascular compartment during M-AHT.

A schematic representation of the key reactions of the gaseous mixture oxygen/ozone on the blood during ex vivo treatment, with the production of the effector molecules responsible for the functional effects on the intravascular compartment during M-AHT, is shown in Figure 2.

Wanting to get more specific it is possible to say that: (i) In red blood cells stimulated by ozone there is a transient increase in the production of intracellular ATP with an increase in glycolysis rate, leading to the stimulation of the Krebs cycle through the production of 3-phosphoglycerate by the 2,3 diphosphoglycerate phosphatase, with changes in the Hb dissociation curve, rising the delivery of oxygen to the tissues [41]; (ii) In mononuclear cells, ozone stimulates immune responses by modulating the transcription factor NFkB and thus it helps to reactivate a depressed immune system [42,43]; (iii) In platelets, ozone stimulates a partial release of growth factor, very useful in patients with chronic wounds that do not heal [22].

Moreover, crucial messengers derive at the late phase from the specific oxidation of only unsaturated fatty acids (the optimal substrate for ozone) present mostly in the plasma albumin. Thus hydroperoxide, but mostly 4-hydroxy-nonenal (4-HNE), which is the terminal compound of lipid peroxidation of n-6-PUFA, forms an adduct with either cysteine or glutathione (GSH) or the Cys34 or the 11 nucleophilic groups of albumin [44]. Therefore, within 5–7 min, the infusion of ozonated blood into the donor patient provokes not only the activation of blood cells but allows the transport of 4-HNE all over the body, particularly the heart, liver, kidneys, lungs, and hypothalamus. Please note that only the use of blood simultaneously taken from the same patient is allowed.

It is now well demonstrated that 4-HNE enters into a great number of cells and reacts specifically with two cysteines present in a protein named Keap-1. This protein normally binds and represses another protein called Nrf2, which represents another normal transcription factor that is usually digested in the proteasome every 20 min. However, when the two critical cysteines (Cys273 and Cys288) of Keap1 have bound 4-HNE, Keap1 releases Nrf2, which is then able to enter the nucleus of many cells. Here it binds to the Antioxidant Response Element (ARE) in the DNA. This most important step allows the activation of some 220 genes which are transcripted into the most important antioxidant enzymes (SOD, GSH-reductase, GSH-transferase, GSH-synthetase, HO-1, catalase, and moreover into phase-2 enzymes crucial for cell detoxification). This simply means that light oxidant stress provokes an intensive modulation of antioxidants of the body [45,46,47].

Thus, a great number of cells in various organs upregulate the synthesis of antioxidants which significantly are able to counteract the excess of reactive oxygen species (ROS) extremely deleterious in metabolic chronic diseases, where chronic oxidative stress is present [48]. It is not the purpose of this article to clarify for the reader all the factors and co-factors that may play a role in balancing oxidative stress. For an exhaustive knowledge of them, please refer to the countless specific writings. The purpose of our article, however, is to investigate in detail the comparative level between the number of molecules of ozone as a paradoxical oxidative stressor and the defensive endowment of antioxidants naturally present in the human blood (e.g., albumin, glutathione, uric acid).

As previously stated, it is good to point out right away that the term ozone therapy includes a multiplicity of methods of administration [10]. We intend to refer to what is commonly known as major autohemotherapy (M-AHT). It can be considered as an ex vivo extra-vascular treatment in a static model of autologous blood to be reinfused within a brief period of time (about 20 min) to the same patient. Ozone therapy is performed by using containers adequate in terms of both ozone-resistant materials and capacity. If glass bottles are used, attention must be paid to the treatment methods, to avoid overpressures that could modify the solubility characteristics and the bubble formation of the ozone itself [49]. Other practical aspects, such as the quantity of blood to be treated, the choice of the anticoagulant and its methods of use, the time, and methods of contact between blood and the gaseous mixture, the speed of re-infusion, even if very important for the standardization of the method, are beyond the scope of this paper. For the sake of clarity, the term “auto-blood transfusion” is sometimes used to signify ozone therapy this ought to be considered incorrect. In fact, autotransfusion is a technique of taking blood from a patient to subsequently re-transfuse it, if necessary, to the same patient. The term autotransfusion identifies the procedures for self-donation of blood, acute normovolemic haemodilution and intra- and/or post-operative blood recovery and washing. For all these treatments, the quantities of blood and times used are quite dissimilar to those used in ozone therapy [50]. On the other hand, ozone therapy is based upon activation of the blood using ozone and implements the minimal stress necessary to stimulate the bodies’ reaction in upregulating the natural antioxidant system. As previously shown, ozone therapy is certainly non-toxic because minimal dosages are used in comparison to the wealth of plasma antioxidants.

## 3. Clinical Applications

### 3.1. An Appraisal of Recent Literature

The therapeutic potential of this treatment is recognizable from the numerous clinical trials (Table 1), some of which are currently available in the literature, especially for COVID-19 treatments [51,52,53].

Moreover, several pilots, but crucial clinical reports in chronic limb ischemia [54,55], age-related macular degeneration [56], chronic obstructive pulmonary disease [57], have already shown the effectiveness of ozone therapy making clear that the ozone dosage must be adequately adjusted. Other pathologies where ozone therapy can be combined with standard therapies are:

- Acute and chronic infectious diseases, particularly due to antibiotic or chemo-resistant bacteria, viruses, and fungi. It must be clear that ozone therapy in itself cannot substitute for antibiotics because both ozone and hydrogen peroxide are exhausted in the glass bottle and are not present in the circulation; however, it is a clinically useful supportive adjunctive therapy [58].
- Ischemic diseases (cerebral and heart ischemia). Anecdotal results seem positive, but they are yet to be validated in randomized clinical trials [59].
- Degenerative disorders: autohemotherapy helps patients in the early phase of senile dementia. On the other hand, it is potentially useful in diabetic retinopathy, retinitis pigmentosa, multiple sclerosis [60,61]
- Pulmonary diseases: emphysema, asthma, and acute respiratory distress syndrome. These affections are becoming the fourth cause of death. Ozonated autohemotherapy performed using low concentrations of ozone dosing reduces the chronic oxidative stress and improves oxygenation thus providing an important clinical adjunct for these patients [45,62].
- Terminal nephropathies are progressively aggravated by chronic oxidative stress and as yet mainstream medicine has not developed the therapeutic means to control or modulate these. Ozone therapy could stabilize this serious dysfunction and improve the quality of life of these patients [63].
- Similarly, in the metabolic syndrome ozone therapy is proving to be very useful well exemplified in patients with type-2 diabetes, also suffering from chronic ulcers with no tendency to heal [64].
- Chemoresistant metastatic cancer and therapy of cancer-related fatigue [65].
- Chronic fatigue syndrome and fibromyalgia, where ozone therapy appears to be useful in most patients [66].
- Sickle cell disease, where ozone therapy appears to be very useful because it procures clinical improvement without adverse effects [67].

### 3.2. Other Modalities of Ozone Administration

As for clinical use of ozone, the systemic application using the modality of the intramuscular injection, known as minor ozonated autohemotherapy (5 mL of blood plus 5 mL of gas with a high ozone concentration, such as 100 µg/mL per mL of blood), used as an autovaccine was considered beyond the aim of the present article [68]. Moreover, the biochemical fundamentals described for systemic administration can also be applied in well-confined anatomical areas. Small amounts of para-vertebral ozone have demonstrated great therapeutic efficacy in the treatment of herniated intervertebral discs. This application can be better understood when compared with other modes of ozone delivery. For example, the loco-regional uses of gaseous ozone in muscle and joint pathologies, lumbosciatica pain, have over time proven to be as effective if not more effective than treatments with conventional medicines, and this without the appearance of side effects, if properly applied [69].

Finally, topical and/or loco-regional applications of ozone and its derivatives are not considered in the present work. Their use as a therapeutic means can be traced to this non-exhaustive list: osteomyelitis, pleural empyema, peritonitis, abscesses with fistulae, bedsores, chronic ulcers, diabetic foot, burns, insect and jellyfish stings, infected wounds, onychomycosis, Candida and Chlamydia infections, Herpes Zoster and Papillomavirus infection, tinnitus, nystagmus, hearing loss and vertigo, trauma, burn injuries and osteonecrosis of the jaw. Ozone and its derivatives increasingly represent an important therapeutic possibility also in both dental [70,71] and veterinary fields [72].

## 4. Molecular Aspects

### 4.1. Antioxidant System

After innumerable M-AHT in patients with various chronic diseases maintained by chronic oxidative stress, it has become imperative to measure how many ozone molecules added to an adequate volume of blood are necessary to elicit a mild but sufficient acute oxidative stress necessary for developing a tolerable acute oxidative stress which will act as the crucial stimulus for reactivating and upregulating the body’s antioxidant system [73]. This concept explains why ozone therapy is becoming an essential integrative system able to restore a normal redox system in numerous clinical applications.

For some time, it has been known that antioxidants and albumin can behave as sacrificial molecules although most of the antioxidants are rapidly reduced by a very efficient recycling system [74] In ozone therapy, this occurs with the infusion of ozonated blood into the patient and this is due to many substances, among which ascorbic acid, GSH, thioredoxin, alpha-tocopherol, lipoic acid, and NADPH. All of these undergo orderly oxidation by a well-coordinated sequence of electron donations [39]. There are other methods for calculating the antioxidant activity of the blood. Variations of the plasma antioxidant activity upon ozone oxidation are measured with specific colorimetric assays [75]. The total antioxidant status (TAS) of plasmas collected by patients can be measured by various methods [76]. Human plasmas of a normal European population have a TAS ranging between 1.28 and 1.83 mmol/L [77], with increased levels in males (men and boys) in comparison with the values found in females (women and girls) [78]. Dysmetabolisms can affect the total antioxidant capacity (TAC) of human plasma, as expected [79].

Ultimately, a well-dosed quantity of ozone can generate an acute, transient, and well-circumscribed stimulus with the consequent formation of effector molecules responsible for the organism’s therapeutic response. About the correctness of the dosage, ozone is produced by modern generators using medicinal oxygen 99.5% as feeding gas. The concentration of ozone produced—generally expressed in µg/mL, with a range usually comprised between 1 and 99 µg/mL—is precisely measured by either a photometer set at 253.7 nm (Hartley band) or using a standard algorithmic calculation method. Under controlled standard time check and validation, both methods are acceptable.

The recommended way to set up ozone therapy is using a gaseous oxygen/ozone mixture starting with the first ozone concentration as low as 10 µg/mL O_2_/O_3_ per ml of blood and the frequency is twice weekly. On every successive week, based on patient feedback about his own clinical status, the ozone gas mixture concentration could be increased stepwise of 5 µg/mL up to a maximum of 40 µg/mL (7th week) per ml of blood. The quantities of whole blood to be treated are around 200 mL. However, the specialist can adapt them in relation to the clinical conditions, the difficulty of sampling and the body mass index. This demonstrates once again that ozone toxicity is not an absolute term, but it depends on the surrounding situation [80].

### 4.2. Practical Considerations

Biological mechanisms elicited by ozone in human blood are an essential part that determines the efficiency of the ozone mass transfer and ozone reactions, even if the area has often been overlooked. In fact, the solubility values that are generally found indicated having no practical application in this context. While oxygen slowly saturates all hemoglobin to Hb_4_O_8_, ozone dissolves and immediately reacts with the common antioxidants and polyunsaturated fatty acids (PUFA). Consequently, owing to the very high reactivity of ozone, within 1–2 min, it oxidizes both the available antioxidants, possibly the albumin cysteine 34 and reacts with either available PUFA.

The peroxidation reaction that happens in the blood, inside either the bottle or the plastic bags, is fundamental because all of the ozone, after the reaction that lasts a maximum of five minutes, disappears but it generates the two critical messengers such as hydrogen peroxide in the group of reactive oxygen substances (ROS) and the hydroxyalkenals in the group of lipoxidation products (LOPs), as previously stated.

Now, it is well known that albumin properties incur some changes under: (i) ischemic attacks associated with oxidative stress [81]; (ii) the production of ROS [82]; (iii) the development of acidosis and diabetes mellitus (DM) and its complications [83].

Human plasma represents almost about 55% of whole blood. Table 2 shows average molecule amounts of readily available antioxidants and PUFA in human plasma [84,85], with respect to ozone molecules at the different concentrations typically used at the therapeutic level. They are approximate values, typically for the lower reference range in adult men.

In such a complex mixture it is not possible to say which is the preferred substrate for ozone activity. Presumably, reaction stoichiometry and relative reaction rate equations depend upon the relative amount of each component and from chemical affinities.

It appears that using only 10 and 15 μg/mL ozone per ml of blood is minimally effective because the number of produced messengers is small. The successive dosages are more effective but nonetheless, the small stress reaction is well accepted by the body. The minimal consumption of reducing agents was confirmed by the minimal reduction of TAS for the first ozone dosage [39]. It appears clear that blood, in comparison to the corresponding plasma, is far less sensitive to ozone oxidation because of the presence of blood cells. The real mechanism of possible self-repair of the cells that have suffered the oxidative insult has not yet been defined, even if the hormesis theory of stress adaptation could represent the cellular response mechanisms to maintain homeostasis and cell survival [86,87].

In simple words, many times after 8–12 autohemotherapy sessions, patients start to feel rejuvenated with more physical and mental strength and this could be due to the increase in the antioxidants. The so far achieved clinical results are amazing and the patient often asks to continue the treatment since there is no toxicity nor side effects even when continuing then therapy for a very long time.

In infectious diseases such as HIV, chronic hepatitis, bacterial diseases, the appropriate antibiotics, or antivirals drugs must be used, and ozone remains an adjuvant therapy. In fact, the idea that ozone is active in blood to destroy viruses and bacteria is totally wrong because the direct decontaminant action of ozone in the blood is paradoxically well protected by the potent antioxidants present in both cells and plasma.

## 5. Unpublished Case Reports

By way of illustration, a case of a male patient (F.R., age 81) affected by subcortical brain ischemia in the period 2010–2015 will be discussed. In the last 3 years, he was treated with ozone therapy (phthalate-free plastic bag at a concentration increased until 40 µg/mL with a volume of blood of 180 mL each time), with a frequency of once one/two weeks. He demonstrated that the pre-conditioning activity of the ozone supplementation [88] was enough for him to manage the SARS-CoV2 infection very well that affected him at the end of 2020, even in the form of a mild-severe form of the disease, with oxygen saturation less than 90%, high fever, diarrhea for some days, cough, dyspnoea, and CT signs of pulmonary insufficiency for COVID-19. Despite all these signs and symptoms, he succeeded in healing from COVID-19 without the use of any medicaments (no corticosteroids, no heparin, no oxygen supplementation) except for ozone therapy once a day for three consecutive days, repeated for two weeks at 15–20 µg/mL concentrations. Today, 6–8 months from the end of the acute phase of the disease, he has a very good immunologic answer to the coronavirus antigen and even after one dose of the two expected vaccination his antibody test is near the superior positive limit. Moreover, he feels very well, he seems to be younger than his age, does heavy both physical and intense and continued intellectual activities. Thus, if someone desires to use ozone therapy in infectious diseases, it must be understood that ozone is useful only as an immune modulator, increasing both the speed and the volume of the immune reaction by increasing the number of the T-lymphocytes, and in the case of viral infective disseminated diseases increasing the CD8+ T-lymphocytes subpopulation. Such a phenomenon follows a helpful vascular endothelial intervention, most likely with the involvement of the corresponding glycocalyx, as evidenced in many other cases [89]. In fact, the glycocalyx that covers the vascular endothelium at the pre-post capillary level plays a fundamental role in the uptake and migration of these CD8+ antiviral cytotoxic lymphocytes by extravascular diapedesis [90].

Analogously, ozone therapy has no direct antitumoral activity in metastatic cancer because neoplastic cells are resistant. On the other hand, it could have positive effects as an adjuvant therapy where there is an analogous CD8+ lymphocytes cytotoxicity against tumoral cells.

In this regard, the case of a female patient is significant (L.S., age 56) infected with HIV from the age of 22 and now suffering from 10 years with Kaposi’s sarcomas in widespread locations (uterus, ovary, intestine, bladder), and very low CD8+ values at the beginning of the therapy. After two years of regularly systemic ozone treatment once every one/two weeks (with a dosage of 20–25 µg of ozone per ml of oxygen), now the patient has no higher value of tumor markers, no metastases discovered with PET, and normal circulating CD8+.

These reports clearly show how important it is to know exactly the mechanisms of action and the necessary individualization in applying ozone therapy.

## 6. Critical Evaluation of Unconventional Systemic Ozone Administration

As for the systemic ozone administration using ozonated saline, Ringer lactate, dextrose and similar, it is critical to clarify the differences with the use of infusion solutions in which the gaseous ozone was dissolved after insufflation of the oxygen/ozone mixture. Leaving aside the toxicological problems deriving from the potential formation of chemical species because of the oxidation reaction of ozone with the chemical species present [91,92], the question is: how to correctly consider these modalities?

As previously stated, ozone therapy can be considered as an ex vivo extra-vascular treatment in static mode of homologous blood to be re-infused in a short time to the same patient. On the contrary, in the case of infusion of ozonated solutions, it is an in vivo intravascular treatment in a dynamic drop-wise mode. Therefore, the molecular correlations with the blood components are the result of many instant reactions. Ultimately, in these cases, the reaction with whole blood occurs at an intravascular level with predictable foam formation with each drop of solution carrying ozone in a molecular active form that enters the bloodstream.

For the sake of completeness, in the context of systemic ozone therapy, the methods of rectal and transdermal administration are also considered. In fact, the absorption capacity of the rectal ampoule and the skin has long been known. however, as regards the rectal administration by insufflation of a gaseous mixture of oxygen-ozone, the major criticalities are represented by: (i) extreme variability of the contents of the rectal ampoule in relation to the fecal mass and its qualitative–quantitative composition; (ii) direct impact of ozone with the cells of the intestinal epithelium covered only by a minimal layer of rectal mucus, in analogy with what has already been seen about the pulmonary epithelium and the corresponding epithelial lining fluid; (iii) different attitudes to absorption between the various hemorrhoidal venous plexuses; (iv) lack of experimental evidence on the absorption of ozone as such, with the consequent achievement of the circulatory stream and formation of ROS and LOPs, however in an intravascular dynamic mode [25]. Similar considerations can be applied to administration through the skin by using a suitable bath or cabinets in controlled humidity conditions [93].

## 7. Conclusions

The proper ex vivo impact of gaseous ozone on blood constitutes the essential topic of the therapeutic treatment due to the ability to form both reactive oxygen species (ROS) and lipid oxidation products (LOPs). These molecules represent the effector molecules responsible for modulating the therapeutic activity in the body after ozonated blood reinfusion in the same subject. Without claiming to describe in detail the complexity of the resulting mechanisms, this article seeks to validate the practice of ozone therapy as an adjuvant treatment in full compliance with the physiology of the whole organism. Ultimately, among a great variety of somewhat doubtful complementary approaches, ozone therapy understood as M-AHT stands as the optimal method to be properly used in chronic metabolic diseases, complicated by chronic oxidative stress.

Until now, such a therapeutic intervention mainly found its application in private clinics all over the world. It is time for ozone therapy to be applied in public hospitals, providing that practicing physicians have been well informed and have passed accredited courses.

## Figures and Tables

**Table 1:** Clinical trials attributable to major autohemotherapy.

Title	Identifier (Location)
Blood Ozonization in Patients With SARS-CoV-2 Respiratory Failure (CORMOR)	NCT04388514 (Italy)
Oxygen–Ozone as Adjuvant Treatment in Early Control of COVID-19 Progression and Modulation of the Gut Microbial Flora (PROBIOZOVID)	NCT04366089 (Italy)
Indirect Endovenous Systemic Ozone for New Coronavirus Disease (COVID-19) in Non-intubated Patients (Ozono COVID-19)	NCT04359303 (Spain)
Ozone Autohemotherapy for COVID-19 Pneumonia (COVID-19 OZONE)	NCT04370223 (Spain)
Ozone Therapy in the Prevention of COVID-19 Infection	NCT04400006 (Turkey)
Efficacy of Medical Ozone Therapy in Patients With Chronic Hepatitis B (EMOTCHB)	NCT01342185 (China)
Oxygen-ozone Therapy Plus Antibiotic Therapy in the Treatment of Infections Secondary to Implant of Orthopaedic Devices	NCT04787575 (Italy)
Clinical study for ozonated autohemotherapy in the treatment of Novel Coronavirus Pneumonia (COVID-19)	ChiCTR2000030165 (China)
A randomized controlled trial for the efficacy of ozonated autohemotherapy in the treatment of Novel Coronavirus Pneumonia (COVID-19)	ChiCTR2000030006 (China)
Study for improvement of myocardial ischemia-reperfusion injury after perioperative cardiopulmonary bypass by ozone autotransfusion combined with electroacupuncture point therapy	ChiCTR2000029612 (China)
Synergy effects and health regulation effect of oxygen-ozone therapy on systemic lupus erythematosus (SLE)	ChiCTR-IOR-17012802 (China)
Ozone treatment for Acute Ischemic Stroke within 1 w of Symptom Onset: A Prospective, Randomized, Multi-center, Open-label, Parallel control, Comparative Study	ChiCTR-ICR-15007093 (China)
A multicenter randomized controlled trial for ozone autohemotherapy in the treatment of novel coronavirus pneumonia (COVID-19)	ChiCTR2000030102 (China)
The effect of ozone treatment on serum markers in patients with fatty liver disease	ChiCTR-TNRC-11001273 (China)
Effect of Ozone therapy in the treatment of COVID-19	IRCT20191125045492N2 (Iran)
A study of therapeutic effect of blood ozone therapy of severe COVID-19 patients	IRCT20200616047792N1 (Iran)
Investigation of the effects of medical Ozone Autohemotherapy on clinical and paraclinical features of patients with COVID-19	IRCT20190618043923N4 (Iran)
Comparison of the effectiveness of Ozone therapy with conventional therapy in the improvement of visual pathways function in diabetic patients	IRCT20191125045492N1 (Iran)
Ozone therapy and routine medical treatment efficacy on serum level changes of TNF-α and CRP as well as neurological improvement	IRCT20200202046342N1 (Iran)
Ozone Therapy Effect on Multiple sclerosis	IRCT20171105037262N3 (Iran)

**Table 2:** Average molecules/mL of readily available antioxidants and PUFA in human plasma with respect to ozone molecules/mL in the oxygen/ozone gaseous mixture at the different concentrations used at therapeutic level.

Non-Enzymatic Antioxidants and Polyunsaturated Fatty Acids (PUFA) Levels, in Human Plasma (Lower Reference Range, Adult Men)							
Uric acid ~1.10^19^ molecules/mL							
Ascorbic acid ~1.7 × 10^18^ molecules/mL							
Glutathione ~1.1 × 10^17^ molecules/mL							
Albumin ~2.6 × 10^19^ molecules/mL							
Available PUFA ~2.4 × 10^19^ molecules/mL							
[O_3_, μg/mL]	10	15	20	25	30	35	40
No. O_3_ molecules/mL (×10^19^)	1.3	2.0	2.6	3.3	3.9	4.6	5.2

## Data Availability

Data are contained within the article.
